# Supplementary material for: Baseline and Early Predictors of Good Patient Candidates for Second-Line after Sorafenib Treatment in Unresectable Hepatocellular Carcinoma
Source: Cancers (Basel). 2019 Aug 27;11(9):1256. doi: 10.3390/cancers11091256 (PMC6770447; doi:10.3390/cancers11091256)
Supplement: Supplementary file 1 [file cancers-11-01256-s001.pdf]

Baseline and Early Predictors of Good Patient Candidates for Second-Line after Sorafenib Treatment in Unresectable Hepatocellular Carcinoma

Hitomi Takada, Masayuki Kurosaki, Kaoru Tsuchiya, Yasuyuki Komiyama, Jun Itakura, Yuka Takahashi, Hiroyuki Nakanishi, Yutaka Yasui, Nobuharu Tamaki, Chiaki Maeyashiki, Shun Kaneko, Kenta Takaura, Mayu Higuchi, Mao Okada, Wan Wang, Leona Osawa, Shuhei Sekiguchi, Yuka Hayakawa, Koji Yamashita, Nobuyuki Enomoto and Namiki Izumi

Table S1. Backgrounds of patients by Child-Pugh score.

Variables	Child-Pugh Score 5 (*n* = 102)	Child-Pugh Score 6 (*n* = 88)	*p* Value
Age: years, mean ± SD	71 ± 9.3	72 ± 9.6	0.32
Male: *n* (%)	82 (80%)	67 (76%)	0.49
Maximum size of HCC nodule in the liver: mm, median (range in IQR)	35 (19–70)	45 (20–82)	0.15
Number of HCC nodules: 1/2–3/4 or more, *n* (%)	17 (17%)/21 (21%)/64 (62%)	11 (13%)/11 (13%)/66 (74%)	0.45
Tumor burden (Maximum size + Number of HCC nodules): mean ± SD	11 ± 6.2	13 ± 6.4	0.02
BCLC stage: B/C, *n* (%)	46 (45%)/56 (55%)	40 (45%)/48 (55%)	1.0
Presence of MVI: *n* (%)	22 (22%)	23 (26%)	0.50
Presence of metastasis: *n* (%)	45 (44%)	31 (35%)	0.24
Albumin: g/dl, mean ± SD	3.9 ± 0.32	3.2 ± 0.22	<0.001
Total bilirubin: g/dl, mean ± SD	0.83 ± 0.57	0.95 ± 0.46	0.12
Prothrombin time: %, mean ± SD	100 ± 16	94 ± 16	0.01
ALBI predictor, mean ± SD	−2.6 ± 0.32	−1.96 ± 0.24	<0.001
ALBI grade: 1/2, *n* (%)	50/52 (49/51%)	0/88 (0/100%)	<0.001
AFP: ng/ml, median (range in IQR)	82 (6.8–2480)	540 (32–4196)	0.007
PIVKA- II : mAU/ml, median (range in IQR)	209 (45–1350)	470 (61–4292)	0.09

AFP; alpha-fetoprotein, AFP L3 fraction; Lens culinaris agglutinin-reactive fraction of AFP, ALBI; albumin–bilirubin, BCLC; Barcelona Clinic Liver Cancer stage, HCC; hepatocellular carcinoma, IQR; Inter Quarter Range, MVI; major vascular invasion, PIVKA-II; prothrombin induced by vitamin K absence II, SD; standard deviation.

Table S2. Backgrounds of patients by ALBI grade.

Variables	ALBI Grade 1 (*n* = 50)	ALBI Grade 2 (*n* = 140)	*p* Value
Age: years, mean ± SD	70 ± 8.6	72 ± 9.7	0.11
Male: *n* (%)	42 (84%)	107 (76%)	0.32
Maximum size of HCC nodule in the liver: mm, median (range in IQR)	35 (19–70)	45 (20–82)	0.15
Number of HCC nodules: 1/2–3/4 or more, *n* (%)	17 (34%)/21 (42%)/12 (23%)	11 (8%)/11 (8%)/118 (84%)	0.45
Tumor burden (Maximum size + Number of HCC nodules): mean ± SD	9.9 ± 6.4	13 ± 6.2	0.005
BCLC stage: B/C, *n* (%)	21 (42%)/29 (58%)	65 (46%)/75 (54%)	0.62
Presence of MVI: *n* (%)	8 (16%)	37 (26%)	0.18
Presence of metastasis: *n* (%)	24 (48%)	52 (37%)	0.18
Albumin: g/dl, mean ± SD	4.1 ± 0.26	3.4 ± 0.32	<0.001
Total bilirubin: g/dl, mean ± SD	0.60 ± 0.18	0.99 ± 0.57	<0.001
Prothrombin time: %, mean ± SD	105 ± 13	95 ± 16	<0.001
ALBI predictor, mean ± SD	−2.9 ± 0.21	−2.1 ± 0.29	<0.001
AFP: ng/ml, median (range in IQR)	82 (6.8–2480)	540 (32–4196)	0.007
PIVKA- II : mAU/ml, median (range in IQR)	209 (45–1350)	470 (61–4292)	0.09

AFP; alpha-fetoprotein, AFP L3 fraction; Lens culinaris agglutinin-reactive fraction of AFP, ALBI; albumin–bilirubin, BCLC; Barcelona Clinic Liver Cancer stage, HCC; hepatocellular carcinoma, IQR; Inter Quarter Range, MVI; major vascular invasion, PIVKA-II; prothrombin induced by vitamin K absence II, SD; standard deviation.

Table S3. Sensitivity and Specificity to satisfy RESORCE criteria.

Baseline and Δ Albumin	Both Predictors Positive	Either or Both Predictors Positive
Sensitivity	29%	85%
Specificity	90%	37%
PPV	53%	36%
NPV	76%	86%
**Baseline and Δ ALBI**	**Both Predictors Positive**	**Either or Both Predictors Positive**
Sensitivity	47%	93%
Specificity	84%	36%
PPV	54%	37%
NPV	80%	92%

PPV; Positive Predictive Value, NPV; Positive Predictive Value, ALBI; albumin–bilirubin.

Table S4. Sensitivity and Specificity to satisfy RESORCE criteria plus Child-Pugh A5.

Baseline and Δ Albumin	Both Predictors Positive	Either or Both Predictors Positive
Sensitivity	33%	88%
Specificity	91%	42%
PPV	35%	18%
NPV	90%	96%
**Baseline and Δ ALBI**	**Both Predictors Positive**	**Either or Both Predictors Positive**
Sensitivity	63%	100%
Specificity	83%	36%
PPV	35%	18%
NPV	94%	100%

PPV; Positive Predictive Value, NPV; Positive Predictive Value, ALBI; albumin–bilirubin.

Table S5. Sensitivity and Specificity to satisfy RESORCE criteria plus ALBI grade 1.

Baseline and Δ Albumin, and Tumor Burden	Three Predictors Positive	Two or More Predictors Positive
Sensitivity	45%	91%
Specificity	97%	74%
PPV	45%	18%
NPV	97%	99%
**Baseline and Δ ALBI, and Tumor Burden**	**Three Predictors Positive**	**Two or More Predictors Positive**
Sensitivity	73%	91%
Specificity	97%	76%
PPV	62%	19%
NPV	98%	99%

PPV; Positive Predictive Value, NPV; Positive Predictive Value, ALBI; albumin–bilirubin.

© 2019 by the authors. Licensee MDPI, Basel, Switzerland. This article is an open access article distributed under the terms and conditions of the Creative Commons Attribution (CC BY) license (http://creativecommons.org/licenses/by/4.0/).
